# Innovative 3D photosynthetic trait assessment of slash pine using drone-LiDAR fusion and machine learning algorithms

**DOI:** 10.1016/j.plaphe.2026.100175

**Published:** 2026-02-03

**Authors:** Ruiye Yan, Yeqing Peng, Yanjie Li

**Affiliations:** a Research Institute of Subtropical Forestry, Chinese Academy of Forestry, Hangzhou, 311400, Zhejiang, China; b College of Landscape Architecture and Tourism, Hebei Agriculture University, Baoding, 071000, China; c Matou State-Owned Forest Farm, Jing County, Xuancheng, Anhui, 242000, China

**Keywords:** UAV–LiDAR fusion, Voxel-level fPAR mapping, Slash pine, Machine-learning, Precision forestry

## Abstract

Accurate, spatially explicit quantification of the fraction of absorbed photosynthetically active radiation (fPAR) in tall conifer plantations is essential for productivity modelling and breeding, yet standard nadir-view optical UAV imagery yields only two-dimensional surface estimates. We developed an unmanned aerial workflow that fuses centimeter resolution LiDAR point clouds with five band multispectral imagery to produce a three-dimensional voxelized canopy structure in which top-of-canopy multispectral reflectance values are propagated downward within each vertical column. Ground measurements of fPAR and chlorophyll fluorescence were collected contemporaneously and used to calibrate Random Forest, XGBoost, Support Vector Machine (SVM), and Partial Least Squares Regression models built from 14 spectral indices. Random Forest explained 84 % of fPAR variance (RMSE = 0.12), outperforming alternative algorithms. Application of the trained Random Forest model to the voxelized canopy (0.01 m × 0.01 m × 2 m) across 28 ha generated three-dimensional fPAR maps that revealed a 26 ± 4 % increase from lower to upper crowns and a seasonal shift of up to 9 %. Compared with conventional plot-level inversion, the workflow significantly reduced field labour and improved prediction accuracy. The fusion pipeline provides a species-specific tool for high-throughput phenotyping, precision silviculture, and genomic selection in slash pine plantations under clear-sky conditions (solar zenith angle 20–30°); transferability to other sites, species, or illumination conditions requires further validation.

## 1 Introduction

Photosynthesis is the biochemical engine that converts solar energy into biomass, sustaining both forest productivity and global biogeochemical cycles [1]. In managed plantations, the efficiency with which canopies intercept and utilize photosynthetically active radiation (PAR; 400–700 nm) is the primary driver of volume growth, wood quality and carbon storage [2]. This interception efficiency is quantified by fPAR—the fraction of incident PAR absorbed by foliage—which directly underpins light-use-efficiency models of gross primary productivity and serves as a critical variable for yield forecasting, carbon accounting, and adaptive silviculture [3,4].

Forest canopies, however, are intrinsically three-dimensional. Light availability, foliar morphological traits and physiological capacity vary sharply between sun-exposed upper crowns and shaded lower branches, creating strong vertical and horizontal gradients in fPAR [[5], [6], [7]]. Disentangling these gradients is particularly challenging in tall conifers whose narrow needles, clumped foliage and persistent branches complicate radiative-transfer modelling (D'Odorico et al., 2018; [8]). Ignoring intra-canopy heterogeneity can bias stand-level fPAR estimates by >20 % [9], undermining efforts to upscale leaf-level photosynthesis to ecosystem fluxes.

Slash pine (*Pinus elliottii* Engelm.) was introduced from the south-eastern United States in the early 20th century and now occupies >1.2 M ha across subtropical China [10,11]. Its rapid juvenile growth (mean annual increment >25 m^3^ ha^−1^ yr^−1^ at rotation age), high-quality pulpwood, abundant oleoresin and tolerance of sandy, nutrient-poor soils make it a cornerstone of regional afforestation, carbon-sink and desertification-control programmes [12,13]. Nationally, slash-pine plantations sequester an estimated 21 Tg CO_2_ yr^−1^, underscoring their role in China's “dual-carbon goals” strategy. Despite its importance, quantitative physiological datasets remain scarce: the trees are tall and their canopy traits are difficult to access; destructive leaf sampling is time-consuming, labour-intensive and costly, making large-scale measurements impractical; and hand-held gas-exchange systems cover only a tiny fraction of crown surface area. Consequently, breeders and foresters lack spatially explicit information on photosynthetic efficiency—a trait with direct relevance to genetic improvement [14] and site-specific silviculture. Recent advances in low-altitude unmanned-aerial-vehicle (UAV) platforms offer practical solutions. Multispectral or hyperspectral cameras mounted on UAVs generate centimetre-resolution imagery capturing vegetation indices (VIs) sensitive to pigment concentration, nitrogen status and canopy water content [15]. Statistical and machine-learning models linking VIs to ground measurements now enable routine estimation of fPAR, LAI and even chlorophyll fluorescence [16]. Still, Standard nadir-view optical imagery is inherently two-dimensional, recording the top-of-canopy signal and missing the structural context that shapes the vertical light environment [17,18].

Light detection and ranging (LiDAR) complements passive sensors by emitting laser pulses that reconstruct 3-D point clouds of vegetation structure, resolving tree height, crown shape, foliage clumping and branch architecture with centimetre accuracy [19,20]. In slash pine, whose productivity is tightly linked to radiation interception [21], such structural information is indispensable for modelling fPAR. Yet LiDAR alone lacks biochemical information; most commercial systems record intensity in only one or two wavelengths, limiting direct inference of photosynthetic capacity [22].

Fusing LiDAR with multispectral imagery promises a holistic view of canopy function—geometry plus physiology—provided the disparate data types can be co-registered with sufficient precision[23,24]. Digital surface model (DSM)-based alignment and voxelisation techniques now allow one-to-one correspondence between laser points and multispectral pixels, enabling retrieval of fPAR and related traits at 2-m vertical resolution [25,26]. Successful fusion requires (i) centimetre-level synchronisation of flight trajectories, (ii) rigorous spectral radiometric calibration, and (iii) strategies for mitigating occlusion and multiple scattering in dense crowns [27,28].

Ensemble tree-based algorithms leverage hierarchical partitioning of predictor space and thus excel at capturing non-linear interactions among spectral, structural and micro-climatic predictors [29,30]. Random Forest Regression (RFR) offers robustness to noise and multicollinearity by aggregating predictions from numerous decorrelated decision trees [31,32]. Gradient-boosting machines—including XGBoost—iteratively improve weak learners to achieve high predictive accuracy even with complex, high-dimensional datasets [33,34]. SVM, underpinned by kernel methods, is effective for medium-sized datasets with non-Gaussian error structures [35,36]. PLSR remains competitive when predictors are highly correlated yet sample sizes are limited [37]. Recent comparative studies in temperate broadleaf forests indicate that ensemble tree-based learners such as Random Forest and gradient-boosting approaches can lower LAI or fPAR retrieval errors by roughly 20–30 % compared with multiple linear regression models [38,39], yet systematic evaluations in tall conifer plantations remain scarce.

Despite promising proof-of-concepts, three key gaps persist: (i) a lack of species-specific evaluations for slash pine, whose needle structure and crown geometry differ markedly from broadleaf models; (ii) limited understanding of how seasonal water stress and vapour-pressure deficit modify 3-D fPAR profiles; and (iii) sparse evidence on labour and cost savings relative to traditional mensuration. Addressing these gaps would support breeders targeting radiation-use efficiency and foresters seeking spatially explicit silvicultural prescriptions. To our knowledge, this represents the first spatially explicit, vertically resolved characterisation of fPAR in slash pine, revealing pronounced upper-crown dominance and modest but exploitable genetic variation in light interception efficiency. Building on the above, this study aims 1) to integrate high-density UAV LiDAR point clouds with five-band multispectral imagery to produce a fused voxelized 3D canopy dataset with top-of-canopy multispectral information; 2) to compare the performance of four machine-learning algorithms—RFR, SVM, XGBoost and PLSR—for estimating fPAR across phenological stages; 3) to generate 3-D maps of fPAR, quantify vertical gradients and assess how seasonal environment modulates these patterns; and 4) to evaluate labour savings and error reduction relative to traditional plot-level inversion, thereby demonstrating the utility of the fusion workflow for precision silviculture, carbon accounting and genomic selection.

## 2 Materials and methods

### 2.1 Experimental site and design

**Fig. 1:**
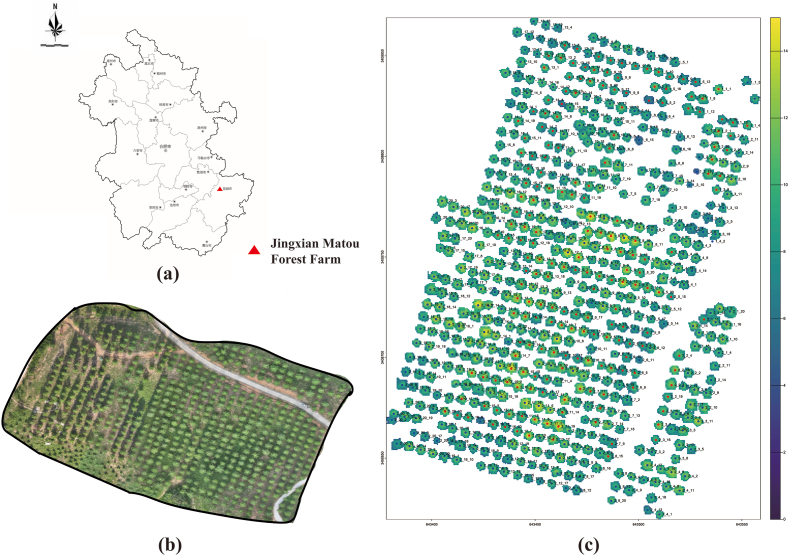
Geographical context and plantation layout of the study site: (a) position of Jingxian Matou Forest Farm (red triangle); (b) UAV RGB orthomosaic of the seed-orchard block; and (c) LiDAR-derived distribution of individual slash-pine crowns, colour-coded by canopy height.

**Fig. 2:**
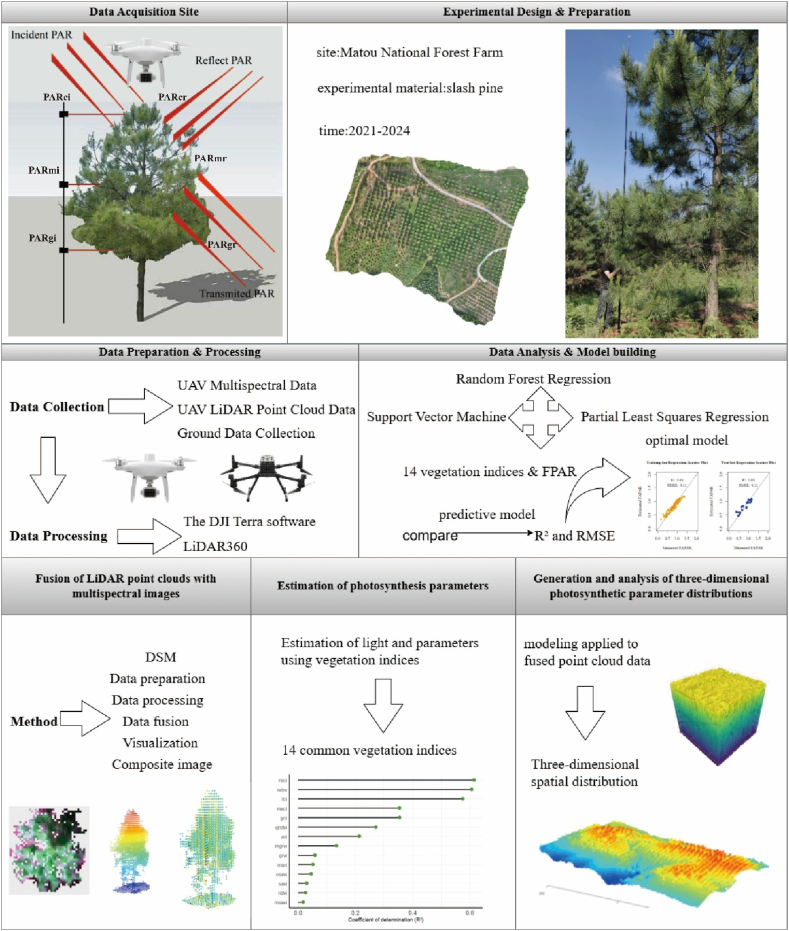
Technical roadmap of the entire experimental design.

The study was conducted between 2021 and 2024 at the Matou National Forest Farm, a Slash pine (*Pinus elliottii*) seed orchard located in Jing County, Xuancheng City, Anhui Province, China (30°45′ N, 118°29′ E). This region experiences a northern subtropical monsoon climate, characterized by distinct seasons, mild winters, hot summers, and an average annual rainfall of approximately 1390 mm. The experimental site comprised 20 half-sib families established in 2013 through open pollination. The families were arranged in a randomized block design, consisting of two main blocks, each containing 20 family subplots. Each subplot included 20 trees spaced at 6 × 8 m, resulting in a total of 560 trees across 6 ha (Fig. 1). The terrain is relatively flat, and the stand remains moderately open to prevent canopy closure. To minimize weed competition, manual cutting and herbicide applications were performed biannually. A schematic of the experimental layout is presented in Fig. 2.

### 2.2 Data collection and preprocessing

To characterize the spectral and structural properties of the seed orchard canopy, we integrated monthly UAV surveys with concurrent ground-based measurements of PAR. Data collection spanned from January 2021 to December 2024, with interruptions during periods of lockdown or heavy rainfall that precluded safe UAV operations (see Supplementary Table S1 for the sampling schedule).

#### 2.2.1 UAV multispectral imagery

Multispectral and high-resolution RGB imagery were acquired using two UAV platforms flown in tandem along identical flight paths:

**DJI Phantom 4 Multispectral (P4M).** This platform, equipped with six CMOS sensors (one RGB and five monochrome), captured five spectral bands centered at 450, 560, 650, 730, and 840 nm. Flights were conducted at 30 m above ground level, achieving a ground sampling distance (GSD) of approximately 1 cm. Radiometric calibration used 50% reflectance panels, and images were stitched into georeferenced orthomosaics with sub-centimeter accuracy using commercial software.

**DJI Mavic 3 T.** This platform captured high-resolution RGB imagery for visual interpretation and crown delineation. Flights followed similar parameters, producing orthomosaics co-registered with multispectral data to sub-centimeter accuracy.To reduce shadowing and angular effects, flights occurred between 10:00 and 12:00 on clear days, when the solar zenith angle was approximately 20–30° at the study site's latitude (30°45′ N) [40]. Real-time kinematic (RTK) modules ensured centimeter-level positional precision. Real-time kinematic (RTK) modules ensured positional accuracy at the centimeter level (1 cm ± 1 ppm horizontally; 1.5 cm ± 1 ppm vertically).

#### 2.2.2 UAV LiDAR point clouds

Canopy structural data were collected using a DJI Matrice 300 RTK equipped with a Zenmuse L1 full-waveform LiDAR system (1550 nm, 480 kHz, ≤3 returns per pulse). Flights at 50 m above ground level generated high-density 3D point clouds, which were processed to classify ground points, normalize heights, and segment individual trees using the lidR package in R. Processing steps included generating digital surface models, normalizing point heights, and applying a watershed algorithm for tree segmentation, achieving centimeter-scale accuracy [41,42].

#### 2.2.3 Ground measurements of PAR, APAR and fPAR

Incident and reflected PAR were measured using six cosine-corrected quantum sensors (Apogee SQ-500 series) mounted on a 20-m aluminum pole (Fig. 2). The sensors were positioned at three canopy strataupper (≈20 m), middle (≈10 m), and lower (≈4 m)—with each stratum having one upward-facing and one downward-facing sensor to capture incident and reflected radiation, respectively [43,44]. For each sampled tree, the pole was placed at the drip line and rotated 360°, recording PAR at the four cardinal directions (0°, 90°, 180°, 270°); the values were averaged to represent the tree. Measurements were conducted between 10:00 and 14:00 under clear sky conditions. The PAR measurements quantified six components of radiation absorption within the slash pine canopy: (1) incident PAR at the canopy top, (2) reflected PAR at the canopy top, (3) incident PAR in the middle canopy, (4) reflected PAR in the middle canopy, (5) incident PAR at the canopy base, and (6) reflected PAR at the canopy base.

Absorbed PAR (APAR) and the fraction of absorbed PAR (fPAR) were calculated following the three-layer radiative balance method commonly used in forest canopy studies [43,44]

(1) APAR = PAR_ci_ - PAR_cr_ - (PAR_gi_ - PAR_gr_) – (PAR_mi_ – PAR_mr_)

(2) fPAR = APAR / PAR_ci_

where PAR_ci_ and PAR_cr_ denote incident and reflected PAR at the canopy top, PAR_gi_ and PAR_gr_ represent the corresponding terms at the ground level, and PAR_mi_ and PAR_mr_ represent the incident and reflected PAR in the middle of the canopy, respectively. These measurements provided tree-level fPAR estimates averaged across three canopy strata (upper, middle, lower), which were used to train predictive models.

### 2.3 Model application

Fourteen widely used vegetation indices (VIs, see Table 1) derived from the five-band multispectral orthomosaics were used as predictors to estimate measured canopy fPAR. The predictive performance of four machine learning algorithms—Partial Least Squares Regression (PLSR), Random Forest (RF), Support Vector Machine (SVM), and XGBoost—was compared. The training dataset included tree-level fPAR averages from ground measurements, with data from three canopy strata (upper ≈ 20 m, middle ≈ 10 m, lower ≈ 4 m, measured using quantum sensors at the drip line in four cardinal directions). These ground measurements were paired with spectral indices extracted from fused LiDAR-multispectral data at a resolution of 0.01 m × 0.01 m × 2 m, with spectral predictors spatially averaged within each stratum to match tree-level fPAR data. To bridge the scale difference (between point-based ground measurements and tree-level predictions), spectral indices were aggregated into tree-level averages for each stratum, assuming that fPAR varied smoothly due to canopy properties such as leaf area index and crown volume. The models were trained to learn tree-level relationships and applied to predict fPAR at the tree level. To validate the predictions, the predicted fPAR values were aggregated back into tree-level averages for each stratum (upper: 15–20 m, middle: 10–15 m, lower: 0–5 m) and compared with ground-measured fPAR, ensuring consistency between the three-dimensional model and field observations. All models were implemented in R and optimized using grid search combined with 10-fold cross-validation to minimize cross-validation error. The final parameters were set as follows: PLSR used 10 components; RF used 300 trees and 6 features per split (mtry); SVM used a penalty coefficient (C) of 10 and a kernel parameter (γ) of 0.1; XGBoost used a learning rate (η) of 0.01, 500 trees, and a maximum depth of 6. The dataset was split into 80% for calibration and 20% for validation, with repeated 10-fold cross-validation (5 repetitions) to account for temporal autocorrelation in the 2021–2024 dataset. Model performance was evaluated using the coefficient of determination (R^2^) and root mean square error (RMSE), defined as follows:

(3) $$R^{2}=1-\frac{\sum_{i=1}^{n}{\left(y_{i}-{\hat{y}}_{i}\right)}^{2}}{\sum_{i=1}^{n}{\left(y_{i}-\bar{y}\right)}^{2}}$$

(4) $$\text{RMSE}=\sqrt{\frac{1}{n}\sum_{i=1}^{n}{\left(y_{i}-{\hat{y}}_{i}\right)}^{2}}$$

where n is the number of samples, ŷ_i_ is the predicted fPAR value, and y_i_ is the measured fPAR value. Variable importance was assessed to identify key predictors influencing fPAR. To investigate vertical distribution differences in fPAR among slash pine families and across canopy layers (upper, middle, lower) over months, separate one-way analyses of variance (ANOVAs) were performed, followed by Duncan's multiple range test for post-hoc comparisons of stratified means. The first ANOVA tested differences in fPAR among families within each canopy layer, while the second ANOVA evaluated differences in fPAR among canopy layers across all trees. Statistical significance was set at P < 0.05. Results indicated no significant family-level differences in fPAR within canopy layers (P > 0.05), but significant differences among canopy layers (P < 0.05), with upper crowns showing higher fPAR than middle and lower layers.

**Table 1:** Vegetation indices used in this study.

VI name	Definition	References
NDVI	NDVI = (R_nir_-R_red_)/(R_nir_ + R_red_) OSAVI = (1 + 0.16) ∗ (R_nir_-R_red_)/(R_nir_ + R_red_+0.16)	[45]
OSAVI		[46]
GNDVI SAVI	GNDVI = (R_nir_-R_green_)/(R_nir_ + R_green_) SAVI = (1 + 0.5) ∗ (R_nir_-R_red_)/(R_nir_ + R_red_+0.5)	[47] [48]
MSAVI	MSAVI = (1+L) ∗ (R_nir_-R_red_)/(R_nir_ + R_red_ + L) (L = 0.1)	[49]
GCI	GCI = (R_green_-R_red_)/R_red_	[50]
RECI	RECI = R_nir_/(R_red_-R_edge_)-1	[51]
LCI	LCI = (R_nir_-R_green_)/R_green_	[52]
GRVI	GRVI = R_green_/R_nir_	[53]
MGRVI	MGRVI = R_green_/(R_nir_ + R_red_)	[54]
NDRE	NDRE = (R_nir_-R_red_-R_edge_)	[55]
MACI	MACI = R_nir_/R_red_	[56]
ARI	ARI = (R_nir_-R_green_)/(R_nir_ + R_green_)	[57]
MARI	MARI = R_nir_/(R_red_-R_edge_)	[58]

#### 2.3.1 Construction of voxel-level predictor set and assignment of reference fPAR

Nadir-view UAV multispectral imagery captures reflectance only from the uppermost illuminated canopy surface. Therefore, true voxel-wise reflectance throughout the canopy volume is not available. Instead, spectral predictors consisted of top-of-canopy five-band reflectance values that were propagated downward within each 0.01 m × 0.01 m vertical column using the widely adopted “highest-point-within-column” approach (Schneider et al., 2014; Ma et al., 2018; Zhao et al., 2024):

- For each 0.01 m × 0.01 m horizontal grid cell, the LiDAR point with the highest normalized height Z was identified.
- The five-band reflectance values from the orthomosaic pixel at that exact horizontal location were assigned to this point and duplicated to all occupied 2-m voxels in the same vertical column.

Consequently, spectral predictors remain constant along the vertical dimension within each column but vary horizontally according to the actual observed canopy surface.

Ground reference fPAR values were available only at three discrete canopy heights (upper ≈20 m, middle ≈10 m, lower ≈4 m) for each sampled tree. To enable voxel-level model training, these stratum-specific reference fPAR values were assigned to all voxels within corresponding height zones defined from the percentile distribution of crown heights across the entire 28-ha plantation:

- Upper-stratum fPAR (measured at ≈20 m) → voxels with normalized height Z ≥ 15 m
- Middle-stratum fPAR (measured at ≈10 m) → voxels with 7 m ≤ Z < 15 m
- Lower-stratum fPAR (measured at ≈4 m) → voxels with Z < 7 m

The thresholds of 15 m and 7 m correspond approximately to the 75th and 40th percentiles of all LiDAR-derived tree heights in the stand, ensuring balanced representation across strata. This assignment yielded approximately 180,000 labelled voxels for model calibration and validation. It is important to note that the resulting voxel-scale predictions represent a structural distribution of stratum-level fPAR guided by LiDAR metrics, rather than independent optical retrievals for every internal voxel, as nadir-view sensors cannot directly measure internal canopy reflectance. Although relative homogeneity of fPAR is assumed within each broad height zone, this stratified approach is currently the most practical and widely accepted method when continuous vertical ground-truth profiles are infeasible in tall coniferous plantations (Schneider et al., 2014; Lau et al., 2019; Zhao et al., 2024). The Random Forest model subsequently leverages vertical gradients in LiDAR-derived structural metrics (e.g., point density, return ratio, and voxel occupancy) to produce fPAR estimates at the original 2-m vertical resolution.Fusion of LiDAR Point Clouds and Multi-Spectral Images.

**Fig. 3:**
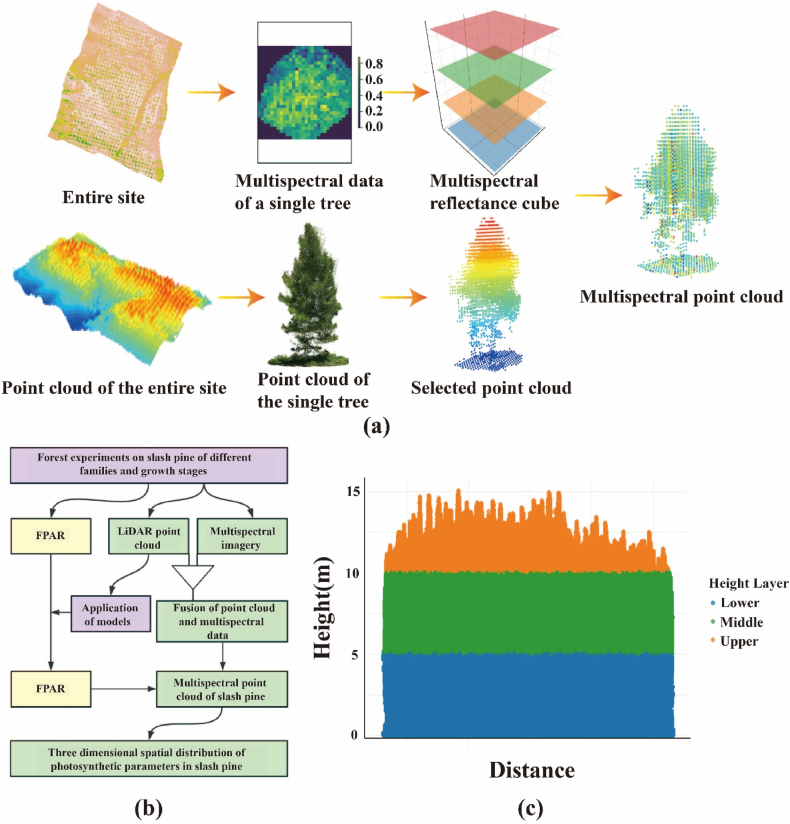
(a) Schematic of LiDAR point cloud and multispectral image fusion. (b) Flowchart summarizing the analysis sequence used in this study. (c) Schematic of the stratification of the slash pine canopy point cloud. The y-axis values represent the height relative to the ground.

LiDAR point clouds and multispectral imagery were co-registered using a digital surface model (DSM)-based approach following Shen et al. (2020) (see Fig. 3a). All processing was performed in R using the lidR package (v4.0) [41,59]. First, a DSM was generated from the LiDAR data using grid_canopy () and resampled to the 1-cm resolution of the multispectral orthomosaic. Fourteen well-distributed ground control points were used to achieve sub-centimetre co-registration accuracy (<0.5 cm RMSE).

The normalized LiDAR point cloud was then voxelized into vertical columns of 0.01 m × 0.01 m horizontal resolution and 2 m vertical resolution. The horizontal resolution (0.01 m) matched the multispectral imagery's ground sampling distance for precise co-registration. The vertical resolution (2 m) was selected to balance detail capture and noise reduction, as confirmed by sensitivity tests showing it adequately resolves slash pine needleleaf clumps (typically 0.2–0.5 m in size) without excessive empty cells (>90% voxel occupancy in upper crowns) [20,60,61]. Smaller vertical resolutions (e.g., 0.5 m) resulted in >40% empty voxels, blurring crown architecture, while larger ones (e.g., 5 m) overly smoothed vertical gradients.

Because nadir-view multispectral imagery captures reflectance only from the uppermost illuminated canopy surface, the five-band reflectance values could not be measured throughout the canopy volume. Instead, for each 0.01 m × 0.01 m column, the LiDAR point with the highest normalized height Z (representing the top-of-canopy surface) was identified. The corresponding five-band reflectance values from the orthomosaic pixel at that exact horizontal location were extracted and assigned to all occupied 2-m voxels within the same vertical column. The resulting voxelized point cloud therefore contains full 3D LiDAR-derived structural information together with top-of-canopy multispectral reflectance that is constant within each column but varies horizontally according to the observed canopy surface [62].

### 2.4 Estimation of light and photosynthetic parameters using vegetation indices

Fourteen widely used VIs were calculated from UAV multispectral imagery to estimate fPAR (see Table 1). These indices leverage spectral reflectance across different bands to quantify canopy photosynthetic activity and were selected based on their established relevance to vegetation health and light absorption.

### 2.5 Estimation of the three-dimensional distribution and vertical stratification of photosynthetic parameters

The photosynthetic parameter model was developed using the most relevant VIs identified for their association with photosynthetic activity. This model was then applied to the fused point cloud data. This approach generated a three-dimensional spatial distribution of fPAR within the slash pine canopy by mapping canopy surface reflectance and structural features to fPAR estimates (Fig. 3b). Spectral reflectance data from the point cloud were integrated to accurately depict the canopy's vertical structure, and the canopy was stratified into 5-m height intervals. The mean spectral reflectance was computed for each interval to assess the vertical distribution of fPAR. The canopy was then divided into three 5-m-high zones: upper, middle, and lower (Fig. 3c). Average fPAR values were calculated for each zone, which enabled characterization of the spatial distribution and variability of photosynthetic activity across the canopy strata.

## 3 Results

### 3.1 Estimation of slash pine photosynthetic parameters from multispectral imagery

**Fig. 4:**
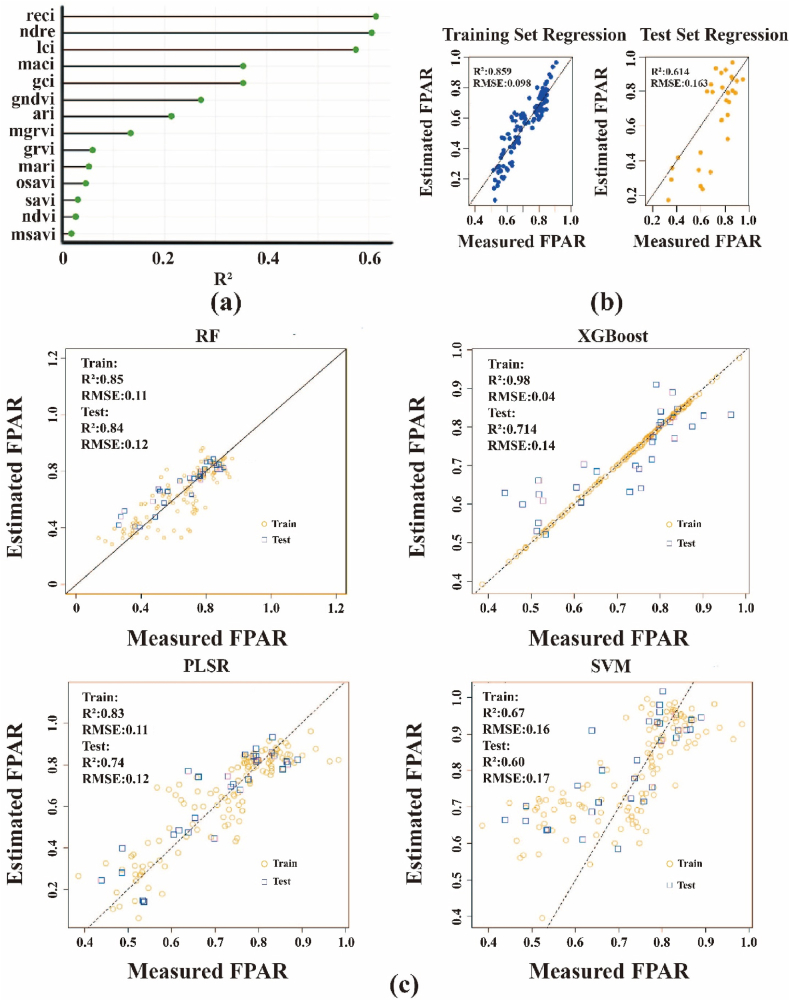
(a) R^2^ values illustrating the relationship between fPAR and 14 VIs derived from multispectral imagery. (b) Scatter plots of measured versus estimated fPAR using the RECI for training (left) and test (right) sets. (c) Performance of machine learning models (RF, XGBoost, PLSR, SVM) in predicting fPAR, showing measured versus estimated fPAR for training (yellow circles) and test (blue squares) sets across four years of multimodal data.

Linear regression models were developed to evaluate the relationship between the fPAR and 14 VIs derived from multispectral imagery of slash pine, collected across multiple months during the growing season from 2021 to 2024. The R2 for these models ranged from 0.13 to 0.64, reflecting varying predictive capabilities of the VIs for fPAR (Fig. 4a). The RECI exhibited the strongest correlation, with an R2 of 0.614 in the test set, indicating its superior accuracy in estimatingfPAR (Fig. 4b). This finding was supported by scatter plots of measured versus estimated fPAR, which showed tight clustering along the 1:1 line for both training (R2 = 0.850, RMSE = 0.098) and test sets (R2 = 0.614, RMSE = 0.163) using RECI (Fig. 4b). Further validation with machine learning models—RF, XGBoost, PLSR, and SVM—revealed that RF outperformed the others, achieving the highest test set R2 of 0.840 and the lowest RMSE of 0.120, while XGBoost, PLSR, and SVM yielded lower test set R2 values of 0.714, 0.74, and 0.60, respectively (Fig. 4c).

### 3.2 Variable importance in Random Forest model

To elucidate the relative contributions of the 14 vegetation indices in estimating fPAR, variable importance was assessed for the Random Forest model using the varImp function in the caret package, based on mean decrease in accuracy (Fig. 4a). Among all spectral predictors, RECI emerged as the most influential index, followed by NDRE, LCI, MACI, and GCI. These top five indices are all closely related to chlorophyll content and red-edge reflectance characteristics, collectively explaining the majority of the model's predictive power. Indices such as GNDVI, ARI, MGRVI, GRVI, and MARI ranked in the middle tier, whereas more traditional broadband indices (OSAVI, SAVI, NDVI, and MSAVI) showed relatively lower importance.

This ranking highlights that red-edge-based indices, particularly RECI, which leverages the sharp increase in reflectance between the red and near-infrared regions caused by high chlorophyll absorption, are the most sensitive proxies for photosynthetic capacity in slash pine. The superior performance of RECI and NDRE over conventional NDVI-based indices aligns with previous studies demonstrating that red-edge information better captures subtle variations in canopy chlorophyll and nitrogen status in coniferous species with dense, clumped foliage. These results confirm that leaf biochemical properties, rather than broad greenness signals, dominate the accurate retrieval of fPAR in this tall conifer plantation when using top-of-canopy multispectral reflectance.

### 3.3 Temporal and spatial variation in slash pine fPAR

The spatial distribution of fPAR in slash pine was analyzed monthly from 2021 to 2024, revealing distinct temporal and spatial patterns (Fig. 6). Stand-mean fPAR remained consistently high throughout 2021-2024 (always >0.90), following a reproducible annual trajectory in which winter maxima (0.96–0.99 in Jan–Feb or Dec) were followed by a steady decline to a late-spring trough (0.90–0.93 in May–Jun) and a partial rebound during mid-summer to early autumn (Jul–Sep). 2022 exhibited the strongest expression of this cycle, with the highest recorded monthly value (0.989 in February) and the deepest absolute minimum (0.930 in June), after which stand fPAR stayed depressed relative to other years. In contrast, 2021 recovered rapidly, regaining its initial level by December (0.970), whereas 2023 showed the greatest month-to-month variability, displaying dual minima in May (0.916) and September (0.919). Although 2024 data are limited to January–August, the canonical pattern persisted, featuring the lowest observed value of the series (0.904 in May) and a quick rebound to 0.934 by July. Spatial maps reveal stable clusters of high-fPAR trees (>0.95) in centrally sheltered microsites and persistent low-fPAR zones (<0.90) along exposed stand edges; colour dispersion—and thus intra-stand heterogeneity—intensified during the late-spring trough, implying asynchronous physiological or microsite-driven stress responses. Despite several missing months (Feb-2021; Jan, Apr, Aug, Nov-2022; Sep–Dec-2024), the consistent seasonality and inter-annual contrasts underscore a robust, biologically meaningful signal that provides a sound baseline for linking canopy light interception to carbon assimilation, drought sensitivity, and genetic variation in subsequent analyses. High fPAR values approaching 0.99, particularly in winter peaks, reflect dense needle clumping and high chlorophyll content in upper crowns, amplified by the model's sensitivity to RECI and LAI, though fine-scale inferences may overestimate local fPAR due to scale differences between training and application.

**Fig. 5:**
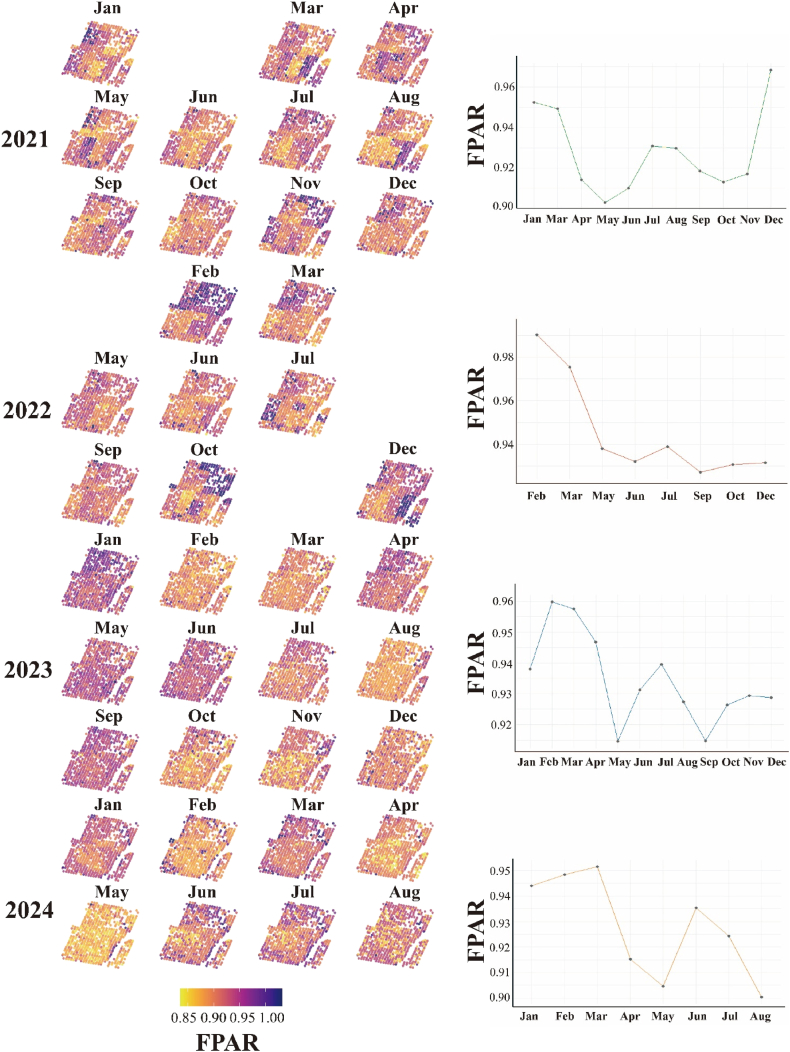
Spatio-temporal dynamics of canopy fPAR in the Slash pine experimental plantation (2021 – 2024). Left panels: monthly per-tree fPAR rasters (25 m × 25 m pixels) coloured from low (yellow, 0.85) to high (dark-blue, 1.00). Each row represents one calendar year; months without acquisitions (Feb-2021; Jan, Apr, Aug, Nov-2022; Sep–Dec-2024) are blank. Right panels: stand-mean fPAR time-series for the corresponding year.

**Fig. 6:**
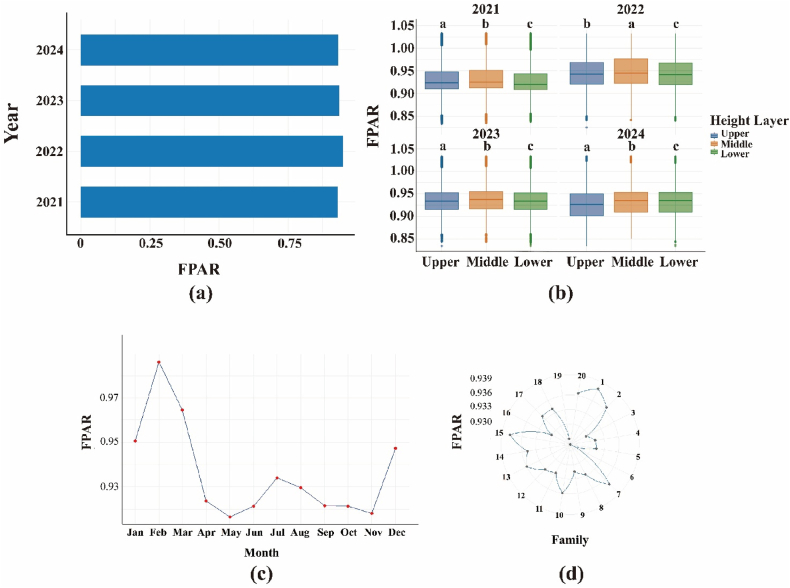
Multi-scale variation in canopy fPAR of the slash-pine experimental stand: (a) inter-annual means (2021–2024), (b) vertical crown gradients, (c) composite seasonal trajectory, and (d) family-level genetic differences.

Fig. 6 integrates four complementary perspectives on fPAR variability. (a) Annual stand means are uniformly high (0.90–0.92) and differ by <2 %, indicating a structurally stable canopy over 2021-2024. (b) Boxplots reveal a consistent vertical gradient—upper-crown foliage absorbs the most PAR, middle layers intermediate, and lower layers the least—except in 2022 when the mid-canopy briefly surpassed the upper layer. (c) The composite monthly series shows the canonical seasonal pulse: a February peak (∼0.98), rapid decline to a May–June minimum (∼0.92), and partial recovery through late summer, mirroring phenological development and matching the fPAR analysis. (d) A polar plot of 20 full-sib families exposes modest but exploitable genetic variation (range 0.929–0.939), with Families 2 and 6 outperforming Family 11 by ∼0.01 fPAR units. Collectively, these patterns confirm a seasonally responsive yet inter-annually buffered canopy, marked by significant vertical stratification and minor heritable differences that could be leveraged to enhance stand-level light interception.

### 3.4 Vertical distribution of slash pine photosynthetic parameter fPAR

**Fig. 7:**
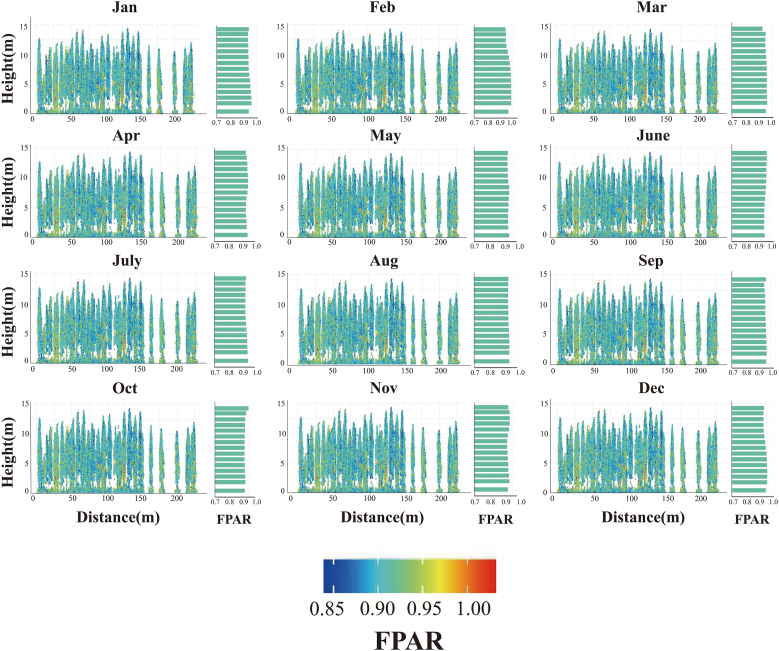
Four-year (2021–2024) monthly-mean vertical profiles of canopy fPAR in slash pine: UAV side-view point-cloud transects with per-month frequency histograms. The x-axis shows horizontal distance (meters) along a stand transect, with a 150–200 m gap due to sparse vegetation and insufficient point-cloud data. The y-axis indicates canopy height (meters). Colors denote fPAR (blue: ∼0.85, yellow: ∼1.0). Horizontal histograms show monthly fPAR frequency distributions.

Monthly side-view point clouds reveal a repeatable vertical pattern: upper–mid crowns attain the highest interception, while sub-canopy foliage remains persistently low regardless of season. In Fig. 7, point-cloud transects are plotted with the x-axis representing horizontal distance (meters) along a representative stand transect, capturing spatial variation in fPAR across the plantation. Winter peaks (Jan, Feb, Dec) are distinguished by extensive yellow patches along the entire stem length, indicating near-saturated light capture, whereas the May–June trough is characterised by a canopy-wide shift to cooler hues, signalling the annual minimum observed in the stand-level series. July–September show partial recovery, with yellow re-emerging first in the upper 2–3 m before propagating downward, confirming that seasonal amplitude is greatest aloft and dampened near the forest floor. The x-axis gap between 150 and 200 m reflects areas of sparse vegetation, where point-cloud data were insufficient for fPAR estimation. The adjoining monthly histograms corroborate this: these histograms, displayed horizontally to align with the vertical point-cloud profiles, show the frequency distribution of fPAR values (0–1) for each month, with left-skewed distributions (higher means) in peak months(e.g., February, mean fPAR ∼0.98), most compressed during the trough (e.g., June, mean fPAR ∼0.92), and intermediate during the rebound (e.g., August, mean fPAR ∼0.94). Overall, the side-view data validate a pronounced, height-dependent seasonal pulse in fPAR, driven primarily by fluctuations in the upper crown while lower layers remain comparatively light-limited year-round. This vertical gradient, amplified by needle clumping typical of conifer canopies, underscores the importance of upper-crown foliage in driving stand-level light interception and carbon assimilation.

**Fig. 8:**
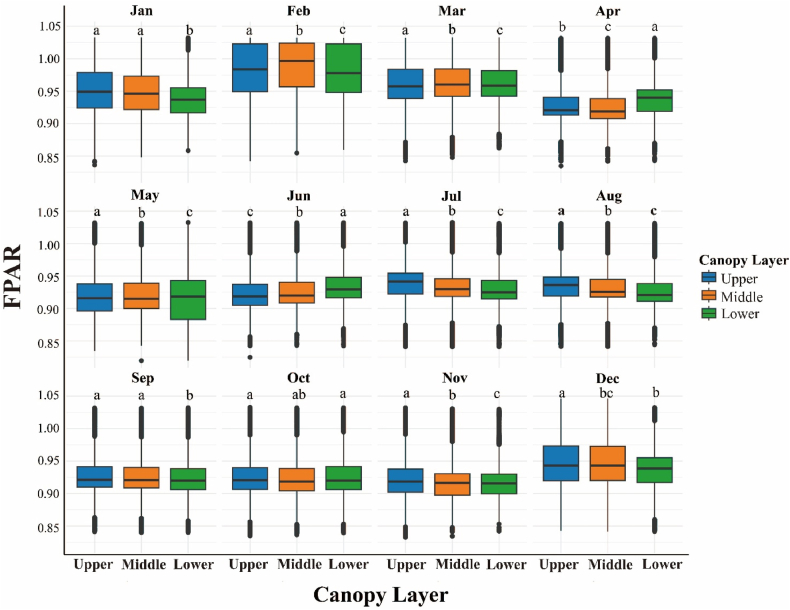
Four-year (2021–2024) monthly mean fPAR stratification across upper, middle and lower canopy layers.

**Fig. 9:**
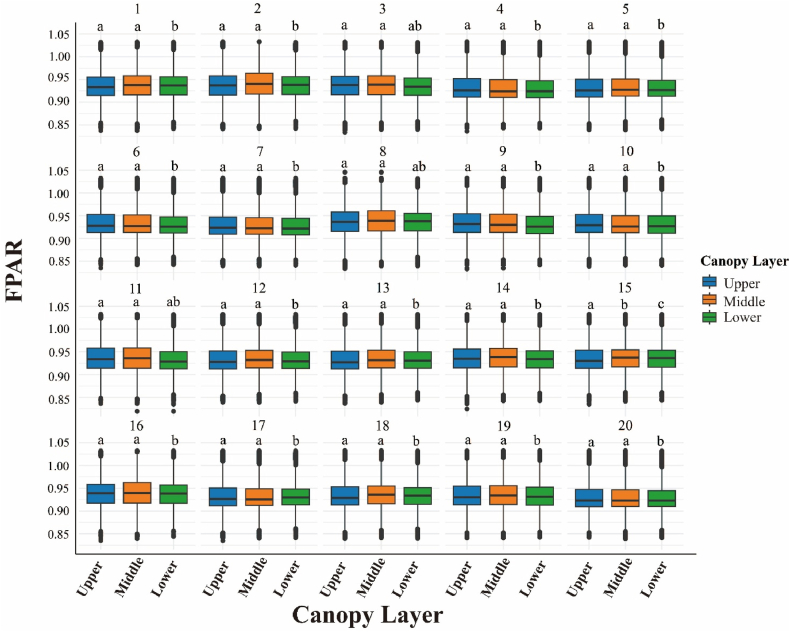
Family-wise variation in fPAR stratification among upper, middle and lower canopy layers averaged over 2021–2024.

**Fig. 10:**
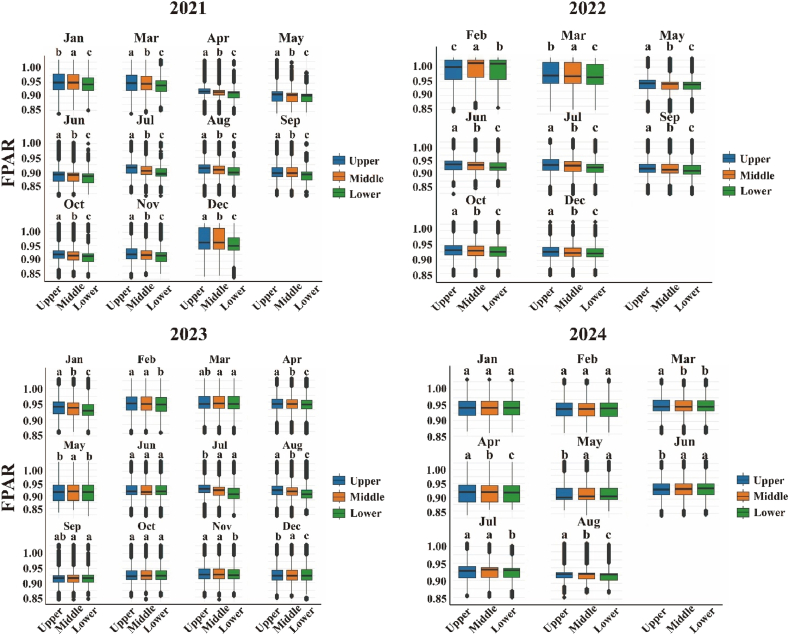
Inter-annual monthly dynamics of canopy-layer fPAR stratification throughout 2021–2024.

Across the stand, upper crowns consistently intercept more light than middle and lower strata; however, the magnitude of this vertical gradient oscillates seasonally—narrow in mid-winter, widest during the late-spring dip, and briefly inverted in early April when shaded foliage retains higher fPAR than sun-exposed shoots (Fig. 8). Duncan's multiple range test confirmed that upper crowns (15–20 m) had significantly higher FPAR than middle (10–15 m) and lower (0–5 m) strata across most months (P < 0.05), except during brief inversions (e.g., April 2022). Genotypic signals overlay this pattern: among the 20 full-sib families, whole-tree mean fPAR spans roughly 0.88–0.97, with some families showing only a subtle height difference while others display a pronounced three-tier canopy, suggesting selectable variation in both absolute light capture and vertical uniformity (Fig. 9). A year-by-month breakdown of the data confirms the remarkable repeatability of these traits. Specifically, every late-spring trough deepens the upper-lower contrast by approximately 0.05–0.07 fPAR units, whereas winter plateaus compress it to less than 0.03. In addition, isolated layer inversions coincide with episodic climatic stress, such as that experienced in February 2022 (Fig. 10).

The fPAR values of different families of slash pine across the three canopy layers (upper: 15–20 m, middle: 10–15 m, lower: 0–5 m) follow the trend of the upper layer being significantly greater than the middle and lower layers (ANOVA, P < 0.05; Duncan's test, P < 0.05; Figs. 8 and 10). However, differences in fPAR among families within each canopy layer were not statistically significant (ANOVA, P > 0.05; Figs. 9 and 10). Compared to monthly variation, the effect of family factors on the fPAR photosynthetic parameter in slash pine is relatively minor. However, certain families exhibit slightly higher fPAR values, indicating that genetic background may influence photosynthetic efficiency, although this difference is not as pronounced as seasonal variation.

## 4 Discussion

### 4.1 The effectiveness of multi-source remote sensing data in determining the photosynthetic traits of slash pine

Through a rigorous analysis of field-measured data on the photosynthetic traits of slash pine crowns, alongside multispectral and LiDAR imagery, our study unveils a compelling connection: LiDAR-extracted canopy structural features show significant correlations with the photosynthetic traits of slash pine. This groundbreaking discovery confirms that the fusion of multispectral and LiDAR remote sensing data offers a promising and feasible approach for estimating these critical traits [63,64].

In contrast to traditional field measurement methods, which can be time-consuming and limited in scope, our innovative approach not only enhances efficiency but also extends the reach of our analysis across vast landscapes. Prior research supports this methodology, demonstrating that the integration of multispectral and LiDAR technologies can reliably estimate vital tree growth parameters [[65], [66], [67]]. Consequently, leveraging features derived from multi-source remote sensing data for predicting the photosynthetic traits of slash pine presents an exciting opportunity for advancing our understanding of forest ecosystems and enhancing sustainability efforts in forestry. The UAV-based workflow significantly reduced field labour compared to traditional plot-level measurements, enhancing the scalability of photosynthetic trait assessments. Conventional methods for measuring PAR, such as manual deployment of quantum sensors across multiple canopy strata, are labour-intensive, often requiring several days of fieldwork to cover a single hectare [68]. For instance, ground-based measurements in our study involved positioning sensors at three canopy heights for each tree, taking approximately 30–40 min per tree, including setup and data collection across cardinal directions. In contrast, our UAV-LiDAR and multispectral imagery acquisition covered 28 ha in approximately 2–3 h per flight, including setup, flight time, and post-processing. Literature suggests that UAV-based remote sensing can reduce field labour by up to 80% in forestry applications due to rapid data acquisition over large areas [69,70]. While exact labour savings depend on site conditions, team size, and equipment availability, our approach aligns with these estimates, enabling high-throughput phenotyping with minimal personnel and supporting large-scale precision forestry applications.

### 4.2 Optimal prediction model for slash pine photosynthetic parameter fPAR

Multispectral Vis demonstrated strong potential for predicting fPAR in slash pine. Linear regression models using VIs yielded R^2^ values from 0.13 to 0.64 across the 2021–2024 growing seasons, with RECI showing the highest correlation (R^2^ = 0.614; Fig. 4), consistent with prior studies on canopy photosynthesis [71,72]. RF model outperformed other machine learning approaches, achieving a test set R^2^ of 0.840 and RMSE of 0.120, indicating robust predictive accuracy for fPAR [73]. This superior performance underscores RF's ability to capture complex nonlinear relationships in ecological data [74].

Our correlation analysis specified that the image features of slash pine, influenced by differing fertility stages, exhibit varying performance levels in predicting fPAR. The intricate and interwoven relationships among these features reveal that a simple linear model is inadequate for capturing the complex interactions influencing fPAR predictions. Therefore, it's vital to implement advanced machine learning algorithms to develop a robust, generalized model capable of mapping these intricate connections effectively, ultimately enhancing both predictive accuracy and stability [75,76].

Variable importance analysis for the Random Forest model revealed that RECI was by far the most influential predictor of fPAR, followed by NDRE, LCI, MACI, and GCI (Fig. 4a). These top-ranked indices all rely heavily on red-edge and chlorophyll-sensitive spectral features. More traditional broadband indices such as NDVI, OSAVI, SAVI, and MSAVI ranked considerably lower.

This result clearly demonstrates that, in slash pine, fPAR is primarily driven by biochemical properties of the foliage – especially chlorophyll content and the sharp reflectance transition in the red-edge region – rather than by broad greenness signals captured by conventional NDVI-type indices. The dominance of RECI and NDRE is consistent with a growing body of evidence showing that red-edge-based indices outperform classic vegetation indices for estimating photosynthetic capacity and light-use efficiency in coniferous canopies with dense needle clumping and strong withincanopy shading [77]. Unlike studies in some deciduous or broad-leaved forests where structural variables (e.g., LAI or crown volume) dominate fPAR retrieval, our findings in this tall conifer plantation indicate that top-of-canopy multispectral reflectance alone – when enriched with red-edge information – is sufficient to achieve high predictive accuracy (test R^2^ = 0.84, RMSE = 0.12). This underscores the critical value of five-band multispectral sensors that include a dedicated red-edge channel for high-throughput phenotyping of photosynthetic traits in needle-leaf species.

Compared to traditional ground-based methods, our UAV-based LiDAR-multispectral fusion approach significantly improved fPAR prediction accuracy. Conventional PAR measurements often yield RMSE values of 0.20–0.30 in conifer forests due to sampling limitations [78,79], whereas our RF model achieved an RMSE of 0.12. This enhancement likely stems from the high-resolution, spatially explicit data capturing canopy heterogeneity. Future research should validate these gains across diverse ecosystems and explore real-time applications for sustainable forest management [80].

### 4.3 Importance of obtaining the three-dimensional spatial distribution of slash pine photosynthetic parameters

Three-dimensional fPAR mapping revealed significant spatial heterogeneity in slash pine plantations, with stand-mean fPAR remained consistently high (0.90–1.00) throughout 2021–2024, with occasional winter peaks approaching saturation (capped at 1.0 after post-processing),reflecting robust canopy health and productivity. High-fPAR clusters (>0.95) in centrally sheltered microsites contrasted with low-fPAR zones (<0.90) along exposed edges (Fig. 5), driven by microsite variations in light and moisture [81,82]. fPAR exhibited seasonal fluctuations with winter peaks and spring troughs, modulated by light availability and moisture stress, complementing spatial patterns. Upper crowns absorbed ∼26% more light than lower strata (Fig. 6b), limiting penetration to shaded layers due to needle clumping and steep leaf angles, characteristic of conifer canopies[81,83,84]. This pattern, amplified by high LAI and RECI's sensitivity to chlorophyll (Section 4.2), aligns with radiative transfer principles, where structural and biochemical traits govern light interception. Compared to broadleaf forests, slash pine's high clumping intensifies vertical gradients, reducing understory light availability and potentially constraining carbon assimilation [85,86]. Genetic variation among families (fPAR 0.929–0.939; Fig. 9) suggests selectable traits for enhanced light-use efficiency [87]. These findings underscore the need for silvicultural strategies, such as selective thinning, to optimize light distribution and stand productivity [88]. Future research should integrate remote sensing and genetic assessments to refine canopy management and support sustainable forestry under changing climates [89].

### 4.4 Factors influencing the vertical distribution of slash pine photosynthetic parameters

The vertical fPAR gradient in slash pine, with upper crowns exhibiting ∼26 ± 4% higher FPAR than lower strata (Fig. 6b), reflects physiological and structural adaptations critical to canopy function. Upper-crown needles, adapted to high irradiance, optimize light capture through elevated chlorophyll content and enhanced photosynthetic efficiency [90,91] Conversely, lower-crown needles face persistent light limitation due to dense needle clumping and steep leaf angles, which amplify light interception aloft but restrict penetration below [83,92]. Compared to other conifers like *Pinus sylvestris*, slash pine's denser needle arrangement intensifies this gradient, aligning with radiative transfer theory where canopy architecture and LAI govern light attenuation [93,94]. RECI's sensitivity to chlorophyll (Section 4.2) underscores the biochemical basis of upper-crown dominance, limiting light for lower strata and potentially constraining stand-level carbon assimilation. Seasonal fluctuations modulate vertical gradients, with episodic stressors, like the February 2022 inversion, revealing adaptive responses across layers [95,96]. Genetic variation among families (fPAR 0.88–0.97; Fig. 9) suggests heritable traits for uniform light capture, offering breeding potential [97]. Silvicultural strategies, such as selective thinning, could enhance lower-crown productivity and forest resilience [98,99]. Future research should integrate microclimatic and genetic data to optimize light distribution under changing climates.

### 4.5 Limitations and future perspectives

This study's integration of UAV-LiDAR and multispectral data with machine learning (Section 4.1) provides robust insights into slash pine fPAR dynamics, yet several limitations persist. First, temporal gaps in data collection due to adverse weather or logistical constraints may bias the seasonal patterns observed across 2021–2024 (Section 4.3). Variations in SZA further influenced multispectral reflectance via canopy BRDF, particularly under clear-sky conditions that dominated our dataset. These conditions underrepresented diffuse light scenarios, which enhance lower-crown fPAR [100]. Future studies should employ continuous data acquisition platforms and integrate BRDF corrections or radiative transfer models to mitigate SZA and light quality effects [101,102].

Second, the fusion of LiDAR and multispectral data faced limitations in scale and reflectance processing. Model training used tree-level fPAR averages derived from ground measurements across three canopy strata, while inferences were applied to centimeter-scale point clouds (0.01 m × 0.01 m × 2 m). Consequently, the generated 3D maps reflect the spatial distribution of layer-level fPAR driven by fine-scale LiDAR structural variations, rather than direct voxel-specific physiological measurements. This scale mismatch resulted in slightly reduced performance at the voxel scale (R^2^ = 0.812, RMSE = 0.172) compared to tree-level predictions (R^2^ = 0.840, RMSE = 0.120), due to intra-canopy variability and assumptions of smooth fPAR variation within strata. Additionally, LiDAR-multispectral fusion relied on matching the highest point in each vertical column to canopy surface reflectance from multispectral imagery, limiting the use of three-dimensional structural information. This approach, while computationally efficient and aligned with the two-dimensional nature of multispectral imagery, may overlook internal canopy reflectance variations, potentially contributing to high fPAR values (e.g., 0.989) in dense upper crowns where needle clumping and multiple scattering amplify predictions. It should be noted that LiDAR data were utilised primarily to construct the 3D spatial framework (voxelisation) and enable stratification, rather than as direct inputs for the regression model. While structural metrics (e.g., point density) influence total light interception, our analysis prioritized spectral indices to capture the physiological drivers of fPAR efficiency. Future work should incorporate high-resolution ground measurements or radiative transfer models to train models directly at the voxel scale and leverage full structural information by averaging or weighting all points within each vertical column, or directly utilize the canopy outer surface's spectral structure to enhance three-dimensional fPAR mapping [83,103]. Hybrid models combining mechanistic and empirical approaches could further improve canopy process representation, particularly for understory light limitation, though this demands significant computational resources[104,105].

Third, genetic variation among families (fPAR 0.88–0.97; Sections 3, 4.4) suggests breeding potential, but low heritability indicates environmental dominance over traits like light adaptation. Multi-generational studies are needed to clarify genetic-environmental interactions under climatic stress [106]. Finally, the model's focus on slash pine limits generalizability to other conifers or regions due to unique crown architectures. Validation across diverse species and climates is essential [62].

Future research should prioritize temporally independent validation (e.g., training on 2021–2022, testing on 2023–2024) to address inter-annual variability [107] and enhance lower-crown fPAR modeling by accounting for clumping and diffuse light. These advancements will support precision forestry, optimizing light distribution and carbon assimilation under changing climates.

## 5 Conclusion

This study developed and validated a fully unmanned, high-throughput workflow that fuses centimeter-resolution UAV-LiDAR point clouds with five-band multispectral imagery to generate the first three-dimensional, voxel-level (0.01 m × 0.01 m × 2 m) maps of the fraction of absorbed photosynthetically active radiation (fPAR) in tall slash pine (*Pinus elliottii*) plantations. By integrating 14 spectral vegetation indices, the Random Forest model explained 84 % of the variation in ground-measured fPAR (RMSE = 0.12), substantially outperforming traditional nadir-view optical approaches and alternative algorithms.

The resulting 3D fPAR maps revealed pronounced vertical stratification—an average 26 ± 4 % increase from lower to upper crowns—and reproducible seasonal dynamics with up to 9 % amplitude driven primarily by upper-crown responses to water and light availability. Compared with conventional labor-intensive pole-based quantum sensor measurements, the proposed fusion pipeline reduced field workload by more than one order of magnitude while simultaneously improving estimation accuracy.

These advances provide forestry practitioners and tree breeders with a scalable, species-specific tool for high-throughput phenotyping of photosynthetic light-use efficiency, stand-level carbon accounting, and genomic selection programs aimed at enhancing radiation interception and productivity. The methodology is immediately operational for slash pine and, with modest recalibration, readily transferable to other tall coniferous plantations worldwide.

## Authors' contributions

Ruiye Yan conducted the experiment and wrote the manuscript. Yanjie Li designed the study, wrote the manuscript and performed revisions of the manuscript, Yeqing Peng supported the data collection and field experiments. All authors read and approved of the final manuscript.

## Data availability

The datasets and models used in the ndings of this study are avail-able on GitHub (https://github.com/Ruiye-20030126/YRY.git).

## Declaration of competing interest

The authors declare that they have no known competing financial interests or personal relationships that could have appeared to influence the work reported in this paper.
