# Supplementary material for: Innovative 3D photosynthetic trait assessment of slash pine using drone-LiDAR fusion and machine learning algorithms
Source: Plant Phenomics. 2026 Feb 3;8(1):100175. doi: 10.1016/j.plaphe.2026.100175 (PMC13109572; doi:10.1016/j.plaphe.2026.100175)
Supplement: Multimedia component 1 [file mmc1.docx]

**Table S1: Remote sensing and field data acquisition dates (2021–2024)**

| **Data Category** | **Year** | **Acquisition Months** |
| --- | --- | --- |
| Multispectral imagery of UAV | 2021 | Jan, Mar–Dec |
|  | 2022 | Jan, Mar, May–Jul, Sep–Oct, Dec |
|  | 2023 | Jan, Mar–Dec |
|  | 2024 | Jan–Aug, Oct–Nov |
| Point cloud of UAV-borne LiDAR | 2021 | Jan, Mar–Dec |
|  | 2022 | Jan, Mar, May–Jul, Sep–Oct, Dec |
|  | 2023 | Jan, Mar–Dec |
|  | 2024 | Jan–Aug, Oct–Nov |
| PAR | 2024 | Apr–Aug |
